# What is the effect of a formalised trauma tertiary survey procedure on missed injury rates in multi-trauma patients? Study protocol for a randomised controlled trial

**DOI:** 10.1186/s13063-015-0733-y

**Published:** 2015-05-13

**Authors:** Gerben B Keijzers, Chris Del Mar, Leo M G Geeraedts, Joshua Byrnes, Elaine M Beller

**Affiliations:** Emergency Physician, Staff Specialist, Emergency Department, Gold Coast Health Service District, Emergency Department, Gold Coast University Hospital, 1 Hospital Boulevard, Southport, 4215 QLD Australia; Assistant Professor, School of Medicine, Bond University, University Drive, Robina, Gold Coast, 4226 QLD Australia; Associate Professor, School of Medicine, Griffith University, University Drive, Robina, Gold Coast, 4226 QLD Australia; Professor of Public Health, School of Medicine, Bond University, University Drive, Robina, Gold Coast, 4226 QLD Australia; Trauma Surgeon, Department of Surgery, VU University Medical Centre, PO Box 7057, 1007 MB Amsterdam, The Netherlands; Griffith Health Institute, Griffith University, Gold Coast Campus, Gold Coast, 4222 QLD Australia; Centre for Applied Health Economics, School of Medicine, Griffith University, Meadowbrook, 4131 QLD Australia; Statistician, Associate Professor, Centre for Research in Evidence-based practice, Bond University, University Drive, Robina, Gold Coast, 4226 QLD Australia

**Keywords:** Missed injury, Patient safety, Tertiary survey, Trauma care

## Abstract

**Background:**

Missed injury is commonly used as a quality indicator in trauma care. The trauma tertiary survey (TTS) has been proposed to reduce missed injuries. However a systematic review assessing the effect of the TTS on missed injury rates in trauma patients found only observational studies, only suggesting a possible increase in early detection and reduction in missed injuries, with significant potential biases. Therefore, more robust methods are necessary to test whether implementation of a formal TTS will increase early in-hospital injury detection, decrease delayed diagnosis and decrease missed injuries after hospital discharge.

**Methods/Design:**

We propose a cluster-randomised, controlled trial to evaluate trauma care enhanced with a formalised TTS procedure. Currently, 20 to 25% of trauma patients routinely have a TTS performed. We expect this to increase to at least 75%. The design is for 6,380 multi-trauma patients in approximately 16 hospitals recruited over 24 months. In the first 12 months, patients will be randomised (by hospital) and allocated 1:1 to receive either the intervention (Group 1) or usual care (Group 2). The recruitment for the second 12 months will entail Group 1 hospitals continuing the TTS, and the Group 2 hospitals beginning it to enable estimates of the persistence of the intervention. The intervention is complex: implementation of formal TTS form, small group education, and executive directive to mandate both. Outcome data will be prospectively collected from (electronic) medical records and patient (telephone follow-up) questionnaires. Missed injuries will be adjudicated by a blinded expert panel. The primary outcome is missed injuries after hospital discharge; secondary outcomes are maintenance of the intervention effect, in-hospital missed injuries, tertiary survey performance rate, hospital and ICU bed days, interventions required for missed injuries, advanced diagnostic imaging requirements, readmissions to hospital, days of work and quality of life (EQ-5D-5 L) and mortality.

**Discussion:**

The findings of this study may alter the delivery of international trauma care. If formal TTS is (cost-) effective this intervention should be implemented widely. If not, where already partly implemented, it should be abandoned. Study findings will be disseminated widely to relevant clinicians and health funders.

**Trial registration:**

ANZCTR: ACTRN12613001218785, prospectively registered, 5 November 2013

**Electronic supplementary material:**

The online version of this article (doi:10.1186/s13063-015-0733-y) contains supplementary material, which is available to authorized users.

## Background

Missed injuries after multi-trauma are a significant health problem, affecting young to middle-aged adults (mainly men). Estimates of incidence are highly variable, but most likely between 1 to 5% of all trauma patients have missed injuries [1,2]. Missed injury is a common quality indicator in trauma care [3,4] and can be the result of several factors, including the prioritisation that takes place during the initial assessment in the Emergency Department (ED), quality of the assessor and institution-specific processes.

The standard primary and secondary surveys by ED, intensive care unit (ICU) and surgical teams have been shown to miss injuries [1,2,5,6]. Performance of a trauma tertiary survey (TTS) has been suggested as a tool to address this problem and minimise the risk of missed injuries. [5] The TTS should follow the episode of emergency care (primary and secondary survey and emergency interventions). It comprises a comprehensive general physical re-examination and review of all investigations, including diagnostic imaging and blood results, within 24 hours [6-8] and again when the patient is conscious, cooperative and mobilised. [5,8,9].

The TTS would be expected to reduce missed injuries and therefore improve trauma care. However, our recent systematic review [2] found sub-optimal evidence to support this.

Among the deficiencies was the substantial variation in outcome definitions for missed injury, leading to a recommendation for a classification (See Table 1). No study reported on missed injury rates after hospital discharge (Type III) or functional (long-term) outcomes. [5,7-14]

**Table 1:** **Missed injury classification** [2]

**Missed injury Type: (delay of)**	**Description**
Type I ≤ 24 hours)	***Before trauma tertiary survey (TTS), or as result of TTS - in-hospital:***
	Injury missed at initial assessment (primary and secondary survey and emergency intervention), but detected within 24 hours, before or through formal TTS.
	(that is, injury missed at initial assessment)
Type II (>24 hours)	***After TTS, in-hospital:***
	Injury missed by TTS, detected in hospital after 24 hours.
	(that is, injury missed at initial assessment *and* TTS,)
Type III (After hospital discharge)	***After TTS, after hospital discharge:***
	Injury missed during hospital stay including TTS,
	(that is, injury missed at initial assessment *and* TTS *and* hospital stay)

A retrospective study [15] in our Australian level II trauma facility found poor compliance to routine TTS and identified a lack of data regarding post-discharge missed injuries. A subsequent, pragmatic, prospective before-and-after study [16] evaluated the implementation of a formalised TTS procedure by measuring missed injury rates during a hospital stay (Type I and II) as well as after discharge (Type III). The in-hospital missed injury rate (Type I and II combined) was similar for both groups (Pre: 3.8% versus Post: 4.8%), as were Type III missed injuries at 1 month (Pre: 13.7% versus Post: 11.5%), and 6 months (Pre: 3.8% versus Post: 3.3%). Although there was a substantial increase in tertiary survey performance (27% versus 42%, *P* < 0.001), this may not have been enough to detect any potential differences in missed injury rates if they exist.

Missed injuries may be minor or only require conservative treatment. Therefore, the focus ideally should be on clinically significant missed injuries. Few studies have defined ‘clinically significant’ *post hoc*, [7,11,12] with most studies not providing any definition. Whilst the incidence of clinically significant missed injuries is unknown, they are more likely to lead to prolonged morbidity or mortality. Although mortality as a result of missed injuries is conceivably rare, our prospective study indicated that approximately 1% of the cohort required a surgical intervention for injuries detected after the hospital discharge. [16].

Current international guidelines such as Early Management for Severe Trauma (EMST) do mention consideration of tertiary surveys, but do not mandate or formalise their implementation. [17] This may be the result of their focus on *early* trauma care, where the tertiary survey by can be conducted up to 24 hours after ED assessment.

There are a number of potential concerns regarding performing a formalised TTS on every multi-trauma patient. It will take more medical and nursing staff time to perform, and may lead to over-investigation. It could lead to over-diagnosis by labelling minor self-limiting conditions with subsequent patient concern or overconsumption of medical resources. As such, it may feed the notion that even minor, self-limiting injuries should always be diagnosed and actively managed. Additionally, re-examination by the same clinician or team may miss the same injury twice due to systematic error or persistent cognitive error. Ultimately, the decision to implement a formalised TTS procedure relies on the assessment of the clinical effect, as well as the value of the clinical benefits, compared to the associated change in costs. However, in order to provide an evaluation of the cost-effectiveness of a formalised TTS, reliable estimates of the effectiveness of a formal TTS is a necessary requirement.

In summary, questions remain regarding the utility and (cost-) effectiveness of the TTS. Although there is a sound rationale for performing formalised TTS, the current data is inconclusive since the quality of data is low and effect size variable. The health care costs to provide this extra layer of safety-net may not prove cost-effective. This protocol specifies the design for a multicentre clinical trial to assess the effectiveness and feasibility of implementing a formal TSS. The results of the study will provide high level evidence that either a TTS should be mandatory in trauma patients, or that we can save resources if no benefit is found.

### Aims and hypotheses

Our primary aim is to determine whether the implementation of a formalised TTS will decrease missed injuries post hospital discharge (Type III injuries) and, as secondary aim, increase injury detection in hospital (Type I and II injuries combined). We further hypothesise that the intervention effect will be maintained and that the intervention would lead to decreased clinically significant missed injuries as judged by a blinded expert panel, decreased hospital and ICU bed days, decreased readmissions to hospital, improved quality of life and decreased time off work. We expect complications of the injuries to be the same, but complications of care to be less. If no difference between injury rates is found, we hypothesise that both arms will be equivalent in resource utilisation (ICU and hospital bed days, investigations ordered, interventions required).

## Methods/Design

This protocol adheres to the SPIRIT statement (www.spirit-statement.org). The Gold Coast Hospital and Health Service Human Research and Ethics Committee approved the study including informed consent processes as outlined under approval number HREC/14/QGC/79.

### Study design

This will be a multi-centre, cluster-randomised, controlled clinical intervention trial. The trial will compare two practices of tertiary survey performance: standard (routine) practice and standard practice with additional formalised trauma tertiary survey completion. This protocol was developed by a small group of experts in the field of tertiary survey in trauma, (including Emergency Physician and Trauma Surgeon) and methodology (Evidence-based medicine, statistics, health economics).

### Study setting

This trial is to be conducted in approximately 16 trauma receiving hospitals across Australia in at least 4 states: Queensland, New South Wales, Victoria and Western Australia. Participating hospitals do not have a dedicated trauma service or formalised process for review of admitted trauma patients.

### Study subjects - patients

All multi-trauma patients admitted to hospital for at least 24 hours will be prospectively identified using and Emergency Department Information System (EDIS or equivalent) and are eligible for recruitment if they meet the inclusion criteria based on previous work [15,16].

### Inclusion criteria

Inclusion criteria as used in previous studies [15,16] are as follows:

1. 16 years and older
2. Admitted for at least 24 hours (since TTS can be performed up to 24 hrs) and any of the following:

- Injuries in two or more body regions
- High impact mechanism (high speed motor vehicle collision, pedestrian versus car, fall from >1.5 meter)
- Chest or abdominal injuries
- Fractured neck of femur under the age of 65 years.

Informed consent for 1, 6 and 12-month telephone follow-up interviews will be obtained from the patient or proxy within 24 hours of enrolment.

### Randomisation

**Figure 1:**
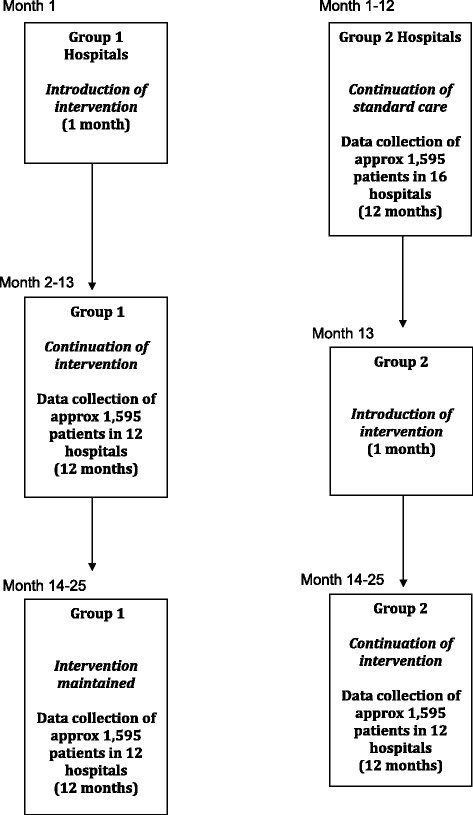
Group allocation, randomisation and timeline.

Participating hospitals (clusters) will be 1:1 randomised to either immediate (Group 1) or delayed (Group 2) introduction of TTS (Figure 1), using computer-generated random number function. Group 1 will implement formalised TTS in the first 12 months, with continuation of formalised TTS in the second 12 months. Group 2 hospitals will continue routine trauma care in the first 12 months followed by implementation of formalised TTS in the second 12 months. A statistician not involved with the data collection will be involved to conduct the randomisation. Results of the randomisation will be communicated to the operations manager of the study, who will inform the participating hospitals of the allocation. All patients of individual hospitals will receive the same treatment arm.

### Informed consent

Individual participants will be asked to consent to follow-up, but not to allocation. Prior to these questions being asked, a brief explanation of the study will be given (patient information sheet); however, we will seek permission to provide limited disclosure to the patient. Specifically, we do not plan to advise them of the study while they are in the ED/hospital, for the following reasons:

1. Patients *will not be individually randomised* and will receive management that is standard practice or standard practice with a formalised TTS.
2. Limited disclosure will help to avoid potentially confounding variables.
3. Risk to the patients in all parts of the study is negligible.
4. Patients are not required to participate in any way whilst in hospital
5. There is little reason to believe that patients would decline participation. In fact, <3% of patients declined participation in our previous study [16].

### The intervention

The primary intervention will be at the hospital level and outcomes will be measured at the patient level. The intervention will consist of the following:

1. The implementation of a formalised TTS form (Additional file 1: Appendix A)*,* used in two previous studies [15,16]). This form prompts documentation for relevant features of history (such as mechanism of injury) and physical examination of relevant body regions. Furthermore, the form prompts for review and documentation of relevant pathology and imaging results. The final part of the form includes a (new) injury list.
2. All medical and nursing staff on trauma admitting wards will attend two education sessions on the rationale and use of the TTS form, provided by a chief or associate researcher in small-group work-shop format.
3. Key stake holder engagement (Hospital executives, Directors of trauma admitting units; Emergency Department, General Surgery, Orthopaedic Surgery, Neurosurgery, Intensive Care Unit) with a clear directive to all levels of medical and nursing staff for compliance with training and education sessions and mandatory TTS form completion as part of routine care within 24 hours of admission.

Standard (existing) care will be at the discretion of the trauma admitting teams of the control hospitals. Standard care is defined as performing a clinical examination based on clinical judgment and without standardised forms. Data collection forms and procedures will be identical for both study arms. A TTS in the intervention phases is defined as completion of the formal TTS form within 24 hours of admission, where during standard care it is defined as a documented tertiary survey as part of a clinical examination within 24 hours of admission. A review of the previously known injuries only is NOT a tertiary survey.

Based on our own descriptive data [15,16], routine TTS are performed in approximately 25% of admitted multi-trauma patients. To truly assess the effect of a formalised TTS on missed injuries, the compliance in the intervention hospitals is aimed to be >90%, but will need to be at least 75% to be deemed an effective implementation of the intervention.

### Time schedule of enrolment

As outlined in Figure 1, the intervention will be introduced over a 1-month period via formalised TTS and education on trauma admitting wards via key stakeholder engagement. In the Group 1 hospitals this will occur at the start of the trial. For the Group 2 hospitals, this introduction period will occur after the 12-month data collection period for routine care (Group 1: TTS implementation → TTS maintenance, Group 2: standard care → TTS implementation). Overall recruitment is expected to take approximately 24 months, which is the estimated time it will take to recruit 6,380 multi-trauma patients, with the final follow-up interviews scheduled at 36 months.

### Data collection

Data collection processes are the same for both Groups. The prospectively identified eligible patients will have their primary and secondary survey performed in the ED. All clinical reviews (including ward rounds) in the first 24 hours on the ward after surgical or interventional radiology intervention will be documented using a previously used [15,16] data collection tool (Additional file 2: Appendix B). Data will be collected by a trained research nurse will include demographic variables, mechanism of injury, Australasian Triage Scale category (ATS) [18] and Glasgow Coma Scale (GCS) on arrival in ED. The Injury Severity Score (ISS) [19] will be calculated after discharge. An ISS score of greater than 15 indicates severe trauma.

Data related to the inpatient admission will include whether a (formalised) TTS is documented during admission and which components of the TTS are performed. A list of injuries, newly detected injuries and changes in management will be collected. A central blinded panel of experts, comprised of at least two emergency physicians and 2 (trauma) surgeons, will class newly detected injuries in-hospital as Type I, Type II or not a new injury (see Table 2). A scripted follow-up telephone interview (Additional file 3: Appendix C) will be conducted at 1, 6 and 12 months after discharge. This follow-up interview will collect data on (Type III) missed injuries after discharge, complications of the injury (parasthaesia, chronic pain) or complications of care (postoperative infections and venous thrombo-embolism), return to pre-injury function, and a validated quality of life (QoL) questionnaire (EuroQoL EQ-5D-5 L [20]).

**Table 2:** **Data Collection**

***Time***	***Data collection process***
Enrolment	A trained research assistant will prospectively review (electronic) medical records for details on all clinical examinations, pathology tests and diagnostic imaging performed in the first 24 hours of admission. A standardised data collection form will be used for all patients. This review will occur between 24 and 48 hours after initial admission. This data is routinely collected data and as such, patient will not be asked to consent to this.
Enrolment	The patient or proxy will be asked to consent for the follow-up at 1, 6 and 12 months. The patient or proxy will be asked to provide at least two contact numbers and will be informed on the detail of these follow up interviews.
Follow-up	Structured interview at 1, 6 and 12 months after hospital discharge. A list of self-reported missed injuries will be adjudicated by an expert panel to classify the in-hospital missed injury (none, Type I or II) and any Type III injury as definite or likely, as well as to determine clinical significance of any missed injury
Follow-up	A trained research assistant will conduct a scripted follow-up interview that will include questions on delayed diagnosis or missed injuries, level of functioning, time off work and quality of life
	*Study measurements*
	Age
	Sex
	Ethnicity
	Occupation
	ISS score.
	Length of Hospital stay, length of ICU stay
	TTS performance and components of TTS (Additional file 1: Appendix A)
	Diagnoses made after 24 hours in hospital, resultant management (active or conservative), diagnostic procedures or tests.
	Predefined complications of care: post-operative infection (wound infection, cathether-related urinary tract infection, pneumonia, sepsis), and venous thrombo-embolism (Deep Vein Thrombosis, Pulmonary embolism)
	Predefined complications of injury: (ongoing or chronic pain, parasthesia, post-concussion syndrome or symptoms)
	Numbers of CT, MRI and ultrasound scans performed
	Details of unplanned attendances and readmissions to hospital
	Patient quality of life at 1, 6, 12 months (EQ-5D-5 L)
	Proportion of usual days of work or study lost at 1, 6 and 12 months
	Missed injury after hospital discharge
	Medical and nursing time used to assess patients in the first 24 hours
	ICD-10 diagnostic and procedure codes
	Australian-Revised Diagnostic-Related Group (AR-DRG) codes
	Community medical services provided
	Primary resource use through Medicare Benefit Scheme (MBS) and the Pharmaceutical Benefit Scheme (PBS) for a 12-month period following the index hospitalizations

If not contactable during initial phone call, up to five attempts will be made. Patients can indicate during follow-up that an injury was missed during their hospital stay. These self-reported injuries will be checked against relevant medical records and imaging reports and defined by the expert panel as missed injury (definitely, likely, unlikely, unknown) or a complication of care or injury. An injury will only be classified as definite or likely (Type III) ‘missed injury’ if there is no evidence (in medical record or radiology report) of the self-reported injury during the hospital stay. The panel will determine the clinical significance of these injuries based on change in management and expected effect on (duration) of morbidity.

Patients who reported a missed injury will be offered appropriate pathways for follow-up. Finally, we will undertake a search of the Death Registry to identify the mortality rate at 12 months post hospital discharge and cause of death.

Appropriate data on the associated cost-implications will be collected during the trial in order to conduct a cost-effectiveness analysis. Items identified that require appropriate measurement and valuation include: medical and nursing time used to assess patients in the first 24 hours, requested advanced imaging type and frequency, ICU length of stay, hospital length of stay, ICD-10 diagnostic and procedure codes, Australian-Revised Diagnostic-Related Group (AR-DRG) codes, patient time-off work, community medical services provided, and re-admissions to hospital.

### Outcome measures

The primary outcome is Type III missed (Post-hospital discharge) injury rate. This study aims to compare the effect of a formalised TTS compared to routine care on missed injury rates after hospital discharge (type III).

Secondary outcomes are:

1. Maintenance of intervention effect (comparing two 12-month periods in Group 1)
2. *During Hospital Stay*
  - In-hospital missed injury rate (Type I and II combined)
  - Proportion of TTS performed
  - Hospital bed days, ICU bed days
3. *During Follow-up (1,6 and 12 months post hospital discharge)*
  - Clinically significant missed injuries (as determined by expert panel)
  - Interventions required for delayed diagnosis (Type I and II) or missed injuries (Type III)
  - Diagnostic imaging usage (CT, MRI, USS)
  - Predefined complications of care and injury (see above)
  - Readmissions to hospital
  - Days off work
  - Quality of life (EQ-5D-5 L)
  - Days off work
  - Mortality

### Statistical analysis and sample size

The primary analysis will be by intention-to-treat (ITT). Per-protocol analyses will also be performed, based on all patients completing 1, 6 and 12 months follow-up. Based on our prospective study [16], comparing routine care with the formalised TTS group, we anticipate a decrease in post discharge (Type III) injuries from 14% to 9%. We foresee this will be accompanied by an increase of in-hospital injury detection (missed injury Type I and II combined) from 4% to 9%.

The sample size for a cluster-randomized trial requires adjustment of the number of patients that a corresponding individually randomized trial would have needed. This is due to variation between the clusters, and results in a correction using the intra-cluster correlation coefficient (ICC) [21]. Using an alpha of 0.05 and a power of 0.80, a sample size of 638 patients per group, with 1-year follow-up completed, would be required if individually randomised. Our previous study [17] achieved a telephone follow-up rate of 40% at 6 months. Extrapolating a similar conservative follow-up rate at 12 months, a total of 3,190 patients will be required (1,595 for each cohort) to answer the primary hypothesis. To separately answer a before-and-after comparison in Group 2 hospitals a similar sample is required. This results in a sample of 6,380 patients, with complete 1-year follow-up in at least 2,552. Since approximately 0.4% [22] of all ED presentations consist of multi-trauma patients, approximately 1,600,000 ED presentations will be required. For an approximate 24-month recruitment period, this equates to 16 hospitals that have an average annual census of 50,000 presentations. Based on this sample size, the maximum ICC that can be present before the power of the study is reduced below 80% is dependent on cluster size and numbers and presented in Table 3*.*

**Table 3:** **Approximate maximum intra-class correlation (ICC) for various cluster sizes and number of intervention hospitals to answer primary hypothesis**

**Number of intervention (and control) hospitals**	**Average size of cluster**	**Maximum ICC to have 80% power to detect 5% difference**
8	400	0.01
15	400	0.02
21	400	0.03
9	300	0.01
15	300	0.02
22	300	0.03
10	200	0.01
16	200	0.02
23	200	0.03

This calculation is consistent with a previous study specifically examining the ICC in trauma receiving hospitals. ICC varied depending on outcome, but the average ICC for blunt trauma the ICC was 0.01 [23]. Assuming an average cluster size of 250 patients, this would mean a total of 3,440 patients would require recruitment (approximately 14 clusters) for the primary hypothesis.

The study is adequately powered for the primary hypothesis, and for several secondary outcomes, such as pre- and post-comparison in Group 2, but not for the pre- and post- comparison in Group 1, measuring maintenance of the intervention effect.

De-identified data will be analysed using SPSS v20.0 software (SPPS Inc, Chicago, IL, USA). For continuous variables, we will use an independent *t*-test and chi-square test will be used to compare differences in proportions. All results will be presented with 95% confidence intervals.

Health economic analyses will include a within trial incremental cost-effectiveness evaluation of formal TTS versus standard care. The outcome measure, incremental cost-effectiveness ratio, directly compares the difference in costs between the formal TTS and standard care with the difference in health related quality of life. Health-related quality of life will be measured in quality adjusted life years (QALYs) derived from changes in EQ-5D-5 L measured at multiple time points. A cost-effective threshold of $AUD 50,000 per QALY will be used to determine whether the intervention was cost-effective and a cost-effectiveness acceptability analysis will be conducted using a range of threshold values from $20,000 to $100,000. A 95% confidence interval around the incremental cost-effectiveness ratio will be estimated using bias-adjusted bootstrapping. In addition, sensitivity analyses and sub-group analyses will be conducted to identify the parameters and patient characteristics that affect the likelihood of the intervention being cost-effective. A *P* value of 0.05 or less will be deemed statistically significant.

### Blinding and choice of outcomes

Blinding the treating staff to the intervention is challenging, but the patient will be blinded since all patients will be receiving a standard care variation (routine versus routine plus formalised TTS) and will not be informed of the specific intervention. However, relatively objective endpoints will be adjudicated by a blinded expert panel and a prospective randomised open-label, blinded end-point (PROBE) methodology can be used [24] and has the advantage that procedures resemble real-world practice and are thus more likely to be broadly generalisable [25,26].

## Discussion

### Outcomes and significance

Missed injury in trauma is a common condition in young adults, with potential to lead to prolonged morbidity. The findings of this study may alter the delivery of international trauma care. If formal TTS is (cost-) effective this intervention should be implemented widely, inform guidelines and change practice. If not, where already partly implemented, it should be abandoned. Study findings will be disseminated widely to relevant clinicians and health funders.

## Trial status

Recruitment has not yet begun because we are awaiting funding.

## Additional files

Additional file 1: Appendix A. - formalised TTS form. Additional file 2: Appendix B. - data collection tool. Additional file 3: Appendix C. - scripted follow-up telephone interview.

## Abbreviations

AR-DRG: Australian-Revised Diagnostic-Related Group
ATS: Australian Triage Scale
CT: computed tomography
ED: Emergency Department
EDIS: Emergency Department Information System
EMST: Early Management for Severe Trauma
GCS: Glasgow Coma Scale
ICC: intra-cluster correlation
ICU: Intensive Care Unit
ISS: Injury Severity Score
ITT: intention to treat
MRI: magnetic resonance imaging
MBS: Medicare Benefit Scheme
PBS: Pharmaceutical Benefit Scheme
PROBE: prospective randomized, open-label, blinded, end-point
QALY: quality adjusted life year
QoL: quality of life
TTS: trauma tertiary survey
USS: ultrasound scan
