# Supplementary material for: What is the effect of a formalised trauma tertiary survey procedure on missed injury rates in multi-trauma patients? Study protocol for a randomised controlled trial
Source: Trials. 2015 May 13;16:215. doi: 10.1186/s13063-015-0733-y (PMC4449594; doi:10.1186/s13063-015-0733-y)
Supplement: Additional file 1: Appendix A. — - formalised TTS form. [file 13063_2015_733_MOESM1_ESM.doc]

**TERTIARY SURVEY FORM**

| 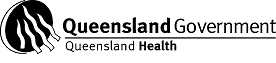  **TERTIARY ASSESMENT OF THE TRAUMA PATIENT**  Gold Coast Health Service District | | | | | | | | | | | | | (Affix patient identification label here)  URN:  Family Name:  Given Names:  Address:  Date of Birth: Sex:  M  F | | | | | | | | | | | | | | | |
| --- | --- | --- | --- | --- | --- | --- | --- | --- | --- | --- | --- | --- | --- | --- | --- | --- | --- | --- | --- | --- | --- | --- | --- | --- | --- | --- | --- | --- |
| ***PHYSICAL ASSESSMENT CONTINUED*** | | | | | | | | | | | | | | | | | | | | | | | | | | | | |
| ***EXTREMITIES***   - ***UPPER & LOWER LIMBS***   - ***PULSES*** | | | - ***Sensation*** | | | | | | | | | | **🗆 *YES* 🗆 *NAD*** | | | | **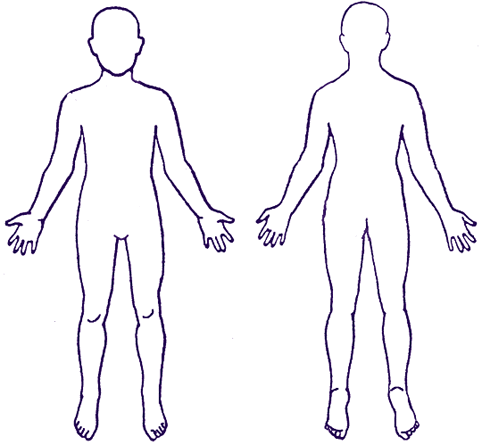**  ***Key:***  ***A = Abrasion C = Contusion L= Laceration***  ***S = Swelling # = Fracture T = Tenderness*** | | | | | | | | | | | |
|  |  |  | - ***Laceration/Abrasion*** | | | | | | | | | | **🗆 *YES* 🗆 *NAD*** | | | |  |  |  |  |  |  |  |  |  |  |  |  |
|  |  |  | - ***Bruising/Swelling*** | | | | | | | | | | **🗆 *YES* 🗆 *NAD*** | | | |  |  |  |  |  |  |  |  |  |  |  |  |
|  |  |  | - ***Tenderness/Fracture*** | | | | | | | | | | **🗆 *YES* 🗆 *NAD*** | | | |  |  |  |  |  |  |  |  |  |  |  |  |
|  |  |  | - ***Motor Function/ROM*** | | | | | | | | | | **🗆 *YES* 🗆 *NAD*** | | | |  |  |  |  |  |  |  |  |  |  |  |  |
|  |  |  | - ***Radial*** | | | | | | | | | | **🗆 *YES* 🗆 *NAD*** | | | |  |  |  |  |  |  |  |  |  |  |  |  |
|  |  |  | - ***Dorsalis Pedis*** | | | | | | | | | | **🗆 *YES* 🗆 *NAD*** | | | |  |  |  |  |  |  |  |  |  |  |  |  |
|  |  |  | - ***Posterior Tibialis*** | | | | | | | | | | **🗆 *YES* 🗆 *NAD*** | | | |  |  |  |  |  |  |  |  |  |  |  |  |
|  |  |  | - ***Colour/Capillary Refill*** | | | | | | | | | | **🗆 *YES* 🗆 *NAD*** | | | |  |  |  |  |  |  |  |  |  |  |  |  |
| ***MANAGEMENT PLAN/ISSUES:*** | | | | | | | | | | | | | | | | | | | | | | | | | | | | |
|  | | | | | | | | | | | | | | | | | | | | | | | | | | | | |
|  | | | | | | | | | | | | | | | | | | | | | | | | | | | | |
|  | | | | | | | | | | | | | | | | | | | | | | | | | | | | |
|  | | | | | | | | | | | | | | | | | | | | | | | | | | | | |
|  | | | | | | | | | | | | | | | | | | | | | | | | | | | | |
|  | | | | | | | | | | | | | | | | | | | | | | | | | | | | |
|  | | | | | | | | | | | | | | | | | | | | | | | | | | | | |
|  | | | | | | | | | | | | | | | | | | | | | | | | | | | | |
|  | | | | | | | | | | | | | | | | | | | | | | | | | | | | |
|  | | | | | | | | | | | | | | | | | | | | | | | | | | | | |
| **🗆 *REVIEW BY TRAUMA SERVICE MEDICAL OFFICER WITHIN 24 HOURS*** | | | | | | | | | | | | | | | | **🗆 *REVIEW BY TREATING TEAM MEDICAL OFFICER***  ***Date:_________________ Time:__________________***  ***Contacted: _____________________________________*** | | | | | | | | | | | | |
| ***PRINT NAME:*** | | | | | | | | | ***SIGNATURE:*** | | | | | | | | | | | | ***DATE:*** | | | | | | | |
| 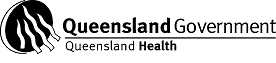  **TERTIARY ASSESMENT OF THE TRAUMA PATIENT**  Gold Coast Health Service District | | | | | | | | | | | | | | (Affix patient identification label here)  URN:  Family Name:  Given Names:  Address:  Date of Birth: Sex:  M  F | | | | | | | | | | | | | | |
| THE TERTIARY SURVEY IS CONDUCTED WITHIN 24 HOURS OF ADMISSION BY THE TRAUMA SERVICE  Version 3 – June 2008 | | | | | | | | | | | | | | | | | | | | | | | | | | | | |
| ***DATE: TIME:*** | | | | | | | | | | | | | | | | ***ADMITTING CONSULTANT:***  ***SPECIALITY:*** | | | | | | | | | | | | |
| ***SURVEY PERFORMED BY:* 🗆 *TCNC* 🗆 *MO*** | | | | | | | | | | | | | | | |  |  |  |  |  |  |  |  |  |  |  |  |  |
| **🗆 *COMPLETE*** | | | | | **🗆 *INCOMPLETE*** | | | | | | | | | | |  |  |  |  |  |  |  |  |  |  |  |  |  |
| **🗆 *PUBLIC*** | | | | | **🗆 *WORKCOVER*** | | | | | | | | | | | **🗆 *PRIVATE HEALTH*** | | | | | | | | **🗆 *DVA*** | | | | |
| ***DATE OF INJURY:*** | | | | | | | ***TIME OF INJURY:*** | | | | | | | | | | | | | ***GCS AT SCENE: E V M*** | | | | | | | | |
| ***DATE OF ARRIVAL:*** | | | | | | | ***TIME OF ARRIVAL:*** | | | | | | | | | | | | | ***GCS ON ARRIVAL: E V M*** | | | | | | | | |
| ***IHT:* 🗆 *Y* 🗆 *N ORIGIN: ____________________*** | | | | | | | | | | | | | | | | ***SECONDARY SURVEY:* 🗆 *Y* 🗆 *N*** | | | | | | | | | | | | |
| ***MECHANISM OF INJURY:*** | | | | | | | | | | | | | | | | | | | | | | | | | | | | |
|  | | | | | | | | | | | | | | | | | | | | | | | | | | | | |
|  | | | | | | | | | | | | | | | | | | | | | | | | | | | | |
|  | | | | | | | | | | | | | | | | | | | | | | | | | | | | |
|  | | | | | | | | | | | | | | | | | | | | | | | | | | | | |
| ***INJURIES:*** | | | | | | | | | | | | | | | | | | | | | | | | | | | | |
|  | | | | | | | | | | | | | | | | | | | | | | | | | | | | |
|  | | | | | | | | | | | | | | | | | | | | | | | | | | | | |
|  | | | | | | | | | | | | | | | | | | | | | | | | | | | | |
|  | | | | | | | | | | | | | | | | | | | | | | | | | | | | |
|  | | | | | | | | | | | | | | | | | | | | | | | | | | | | |
|  | | | | | | | | | | | | | | | | | | | | | | | | | | | | |
| ***ALLERGIES:*** | | | | | | | | | | | | | | | | | | | | | | | | | | | | |
| ***CURRENT VITAL SIGNS*** | | | | | | | | | | | | | | | | | | | | | | | | | | | | |
| ***GCS:E V M*** | | | | | | ***TEMP*** | | | | | | ***BP*** | | | | | ***HR*** | | | | | | ***RR*** | | | ***SATS*** | | |
| ***PATHOLOGY RESULTS*** | | | | | | | | | | | | | | | | | | | | | | | | | | | | |
| - ***FBC*** - ***COAG*** | ***TEST*** | | | | ***Hb*** | | | | | ***PLATLETS*** | | | | | | ***WCC*** | | | ***NEUT*** | | | | | ***INR*** | | | ***APTT*** | |
|  | ***ADMIT*** | | | |  | | | | |  | | | | | |  | | |  | | | | |  | | |  | |
|  | ***CURRENT*** | | | |  | | | | |  | | | | | |  | | |  | | | | |  | | |  | |
| - ***ELFT*** | ***TEST*** | | | ***NA*** | | | | ***K*** | | | ***UREA*** | | | | | ***CREAT*** | | ***LIPASE*** | | | | ***LACTATE*** | | | ***CK*** | | | ***BE*** |
|  | ***ADMIT*** | | |  | | | |  | | |  | | | | |  | |  | | | |  | | |  | | |  |
|  | ***CURRENT*** | | |  | | | |  | | |  | | | | |  | |  | | | |  | | |  | | |  |
| ***TRANSFUSION:* 🗆 *Y* 🗆 *N* 🗆 *RCC* 🗆 *PLT* 🗆 *FFP*** | | | | | | | | | | | | | | | ***ON WARFARIN:* 🗆 *Y* 🗆 *N*** | | | | | | | | ***VITAMIN K GIVEN:* 🗆 *Y* 🗆 *N*** | | | | | |
| - ***URINALYSIS*** | | **🗆 *RCC* 🗆 *LEUCOCYTES* 🗆 *KETONES*** | | | | | | | | | | | | | | | | | | | | | | | | | | |
| ***DVT PROPHLAXIS:* 🗆 *Y* 🗆 *N TYPE: ____________________________________________________*** | | | | | | | | | | | | | | | | | | | | | | | | | | | | |
| ***INVASIVE LINES:* 🗆 *CVL* 🗆 *ARTERIAL* 🗆 *PICC* 🗆  *OTHER*** | | | | | | | | | | | | | | | | | | | | | | | | | | | | |
| ***DRAINS:* 🗆 *ICC* 🗆 *IDC* 🗆 *EVD* 🗆 *OTHER ________________________________________________*** | | | | | | | | | | | | | | | | | | | | | | | | | | | | |

| 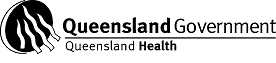  **TERTIARY ASSESSMENT OF THE TRAUMA PATIENT**  Gold Coast Health Service District | | (Affix patient identification label here)  URN:  Family Name:  Given Names:  Address:  Date of Birth: Sex:  M  F | | |
| --- | --- | --- | --- | --- |
| ***PHYSICAL ASSESSMENT*** | | | | |
| ***BODY REGION*** | ***INSPECT & PALPATE*** | | ***FINDINGS*** | ***COMMENTS*** |
| ***HEAD AND FACE***   - ***SCALP*** | - ***Laceration/Abrasion*** | | **🞏 *YES* 🞏 *NAD*** |  |
|  | - ***Swelling/Bruising*** | | **🞏 *YES* 🞏 *NAD*** |  |
|  | - ***Tenderness/Fractures*** | | **🞏 *YES* 🞏 *NAD*** |  |
| - ***FACE*** | - ***Laceration/Abrasion*** | | **🞏 *YES* 🞏 *NAD*** |  |
|  | - ***Swelling/Bruising*** | | **🞏 *YES* 🞏 *NAD*** |  |
|  | - ***Tenderness/Fractures*** | | **🞏 *YES* 🞏 *NAD*** |  |
| - ***EYES*** | - ***Eye Movement*** | | **🞏 *YES* 🞏 *NAD*** |  |
|  | - ***Pupilsize/Reaction/Symmety*** | | **🞏 *YES* 🞏 *NAD*** |  |
|  | - ***Visual Acuity*** | | **🞏 *YES* 🞏 *NAD*** |  |
| - ***EARS*** | - ***Laceration/Abrasion*** | | **🞏 *YES* 🞏 *NAD*** |  |
|  | - ***Discharge*** | | **🞏 *YES* 🞏 *NAD*** |  |
|  | - ***Hearing*** | | **🞏 *YES* 🞏 *NAD*** |  |
| - ***MOUTH*** | - ***Malocclusion/Bite*** | | **🞏 *YES* 🞏 *NAD*** |  |
|  | - ***Teeth Problems*** | | **🞏 *YES* 🞏 *NAD*** |  |
|  | - ***Tounge*** | | **🞏 *YES* 🞏 *NAD*** |  |
| - ***CRAINAL NERVES*** | - ***I - XII*** | | **🞏 *YES* 🞏 *NAD*** |  |
| ***NECK & C-SPINE***   - ***NECK*** | - ***Laceration/Abrasion*** | | **🞏 *YES* 🞏 *NAD*** |  |
|  | - ***Haematoma/S/C Emphysema*** | | **🞏 *YES* 🞏 *NAD*** |  |
|  | - ***Swelling/Bruising*** | | **🞏 *YES* 🞏 *NAD*** |  |
|  | - ***Tenderness*** | | **🞏 *YES* 🞏 *NAD*** |  |
| - ***TRACHEA*** | - ***Central*** | | **🞏 *YES* 🞏 *NO*** |  |
|  | - ***Tracheostomy*** | | **🞏 *YES* 🞏 *NO*** |  |
| - ***C – SPINE*** | - ***Cleared Clinically*** | | **🞏 *YES* 🞏 *NO*** |  |
|  | - ***Cleared Radiologically*** | | **🞏 *YES* 🞏 *NO*** |  |

| 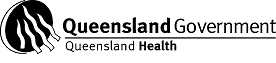  **TERTIARY ASSESSMENT OF THE TRAUMA PATIENT**  Gold Coast Health Service District | (Affix patient identification label here)  URN:  Family Name:  Given Names:  Address:  Date of Birth: Sex:  M  F |
| --- | --- |
|  | |
