# Supplementary material for: What is the effect of a formalised trauma tertiary survey procedure on missed injury rates in multi-trauma patients? Study protocol for a randomised controlled trial
Source: Trials. 2015 May 13;16:215. doi: 10.1186/s13063-015-0733-y (PMC4449594; doi:10.1186/s13063-015-0733-y)
Supplement: Additional file 2: Appendix B. — - data collection tool. [file 13063_2015_733_MOESM2_ESM.doc]

# APPENDIX 1

# **Data Collection sheet – Tertiary Survey Study**

#

# Patient UR: ____________________ (Place pt sticker here)

# _____________________

# _____________________

# **Consent for telephone follow up**

# ❒ **Yes**

# ❒ **No**

# ❒ **Not possible yet**

# Please check contact details on sticker above

# **(alternative phone numbers):**

1.

2.

**Confirm best times to contact:**

1.

2.

# **Date of tertiary survey _______________**

**DEMOGRAPHICS**

# **Date of admission:** ______________

# **2. Date of discharge (if known): ______________**

**3. Age: ______________ years**

# **4.Gender**

#

# ❒ Male

# ❒ Female

#

**5. Triage Category (in ED)**

# ❒ 1

# ❒ 2

# ❒ 3

# ❒ 4

# ❒ 5

**6. Mechanism of trauma**

# ❒ MVA- high Speed (>100 km/hr/ rollover/ejection)

# ❒ MVA – moderate speed, no rollover/ejection)

# ❒ MBA

# ❒ Fall from height > 1meter or 5 steps

# Pedestrian vs car

# Other ________________

**7. GCS (on arrival in ED):______________**

**8. (Initial) Disposition**

# ❒ Surgical ward

# ❒ Orthopedic ward

# ❒ Intensive Care Unit (ICU)

# ❒ Observation Ward

# ❒ Operating Theatre (OT)

# ❒ other:________

#

#

# **Tertiary Survey at 24 hours**

1. **On which admission day has tertiary survey been completed?**

# within 24 hours of admission

# between 24-48 hours of admission

# > 48 hours

# No tertiary survey documented

1. **Who has *performed* the tertiary survey?**

# ❒ Not performed

# ❒ Intern

# JHO/SHO

# Registrar

# Consultant

# Unknown/Unclear

1. **Who has *documented* the tertiary survey?**

# ❒ Not performed

# ❒ Intern

# JHO/SHO

# Registrar

# Consultant

# Unknown/Unclear

**With regards to the documentation of the tertiary survey, have the following components been documented in the chart?**

**Examination**

**– *Current* Vital signs documented?:**

**12. Glasgow Coma Scale (GCS)**

❒ Yes

- No

**13. Temperature**

❒ Yes

- No

**14. Blood Pressure**

❒ Yes

- No

**15. Heart Rate**

❒ Yes

- No

**16. Respiratory Rate**

❒ Yes

- No

**17. Oxygen Saturation**

❒ Yes

- No

**-- Physical Examination documented?**

***-- Head and face***

**18. Scalp**

❒ Yes

❒ No

**19. Face**

❒ Yes

❒ No

**20. Eyes (including visual acuity)**

❒ Yes

❒ No

**21. Ears**

❒ Yes

❒ No

**22. Mouth**

❒ Yes

❒ No

**23. Cranial Nerves**

❒ Yes

❒ No

***-- Neck and C-spine***

**24. Neck**

❒ Yes

❒ No

**25. Trachea**

❒ Yes

❒ No

**26. C-spine (cleared + documented)**

❒ Yes

❒ No

***-- Chest***

**27. Chest Wall (incl Ribs)**

❒ Yes

❒ No

**28. Sternum (incl Ribs)**

❒ Yes

❒ No

**29. Clavicle/Shoulder**

❒ Yes

❒ No

**30. ICC (if present)**

❒ Yes

❒ No

❒ N/A

***-- Abdomen & pelvis***

**31. Abdomen**

❒ Yes

❒ No

**32. Pelvis**

❒ Yes

❒ No

**33. Genitalia**

❒ Yes

❒ No

***-- Back***

**34. General Back**

❒ Yes

❒ No

**35. T-spine and L-spine**

❒ Yes

❒ No

***-- Extremities***

**36. Upper Limbs**

❒ Yes

❒ No

**37. Lower Limbs**

❒ Yes

❒ No

**38. Pulses**

❒ Yes

❒ No

***-- Pathology(documented?)***

**39. Full Blood Count**

❒ Yes

❒ No

**40. Coagulation studies**

❒ Yes

❒ No

**41. UELFTs**

❒ Yes

❒ No

**42. Blood transfusion (documented)**

❒ Yes

❒ No

**43. Plain Radiology (plain films) review documented?**

❒ Yes

❒ No

**44. Who has reviewed Plain Radiology (plain films)?**

❒ Intern

- JHO/SHO
- Registrar
- Consultant
- Radiology registrar
- Radiology consultant

❒ N/A

**45. Advanced Radiology (CT, USS, MRI) review documented?**

❒ Yes

❒ Partially

❒ No

**46. Who has reviewed Advanced Radiology (CT, USS, MRI)?**

❒ Intern

- JHO/SHO
- Registrar
- Consultant
- Radiology registrar
- Radiology consultant

❒ N/A

**47. Are there newly detected injuries as a result of the tertiary survey?**

❒ Yes

- No
- 48. if answered yes to Q47 please specify:

| **Injury** | **Treatment**  **(no Tx, splint, operation, other)** | **Referral**  **(Yes/No, if yes: to who?)** |
| --- | --- | --- |
| 1. |  |  |
| 2. |  |  |
| 3. |  |  |
| 4. |  |  |
| 5. |  |  |

**49. Injury Summary documented?**

❒ Yes

❒ No
