# Supplementary material for: What is the effect of a formalised trauma tertiary survey procedure on missed injury rates in multi-trauma patients? Study protocol for a randomised controlled trial
Source: Trials. 2015 May 13;16:215. doi: 10.1186/s13063-015-0733-y (PMC4449594; doi:10.1186/s13063-015-0733-y)
Supplement: Additional file 3: Appendix C. — - scripted follow-up telephone interview. [file 13063_2015_733_MOESM3_ESM.doc]

Follow up telephone interviews –

Evaluating the Implementation of a Tertiary Survey for

# Admitted Trauma Patients

#

# Patient Study Code: ____________________ Date of interview: _______________

# ❒ 1 month post discharge

# ❒ 6 month post discharge

#

# **Introduce yourself, where you are calling from and ask to speak to the participant themselves if aged 18 years or older or the parent of the study participant (if aged <18 years).**

**If they are not available to come to the phone say:**

“Could you please let me know when might be a more convenient time to call back?”

# **If asked what it is about say:**

# “Name (participant) was admitted to the Gold Coast Hospital about 6 months ago. We phoned a month after discharge to ask questions about how things had gone. During that interview they agreed to be called one final time at 6 months after discharge. Are they available for five to ten minutes, please?”

**If the participant declines to be interviewed, say:**

“Well we hope everything related to your hospital visit went well and that your health is OK now. Thank you for your previous help. Good bye”

**DEMOGRAPHICS (to cross check with chart)**

# **Who completed interview?** **Gender** **Age** (at time of admission)

# ❒ Participant ❒ Male

# ❒ Parent/Caregiver ❒ Female ………years

#

# **INTERVIEW 1-month**

Thanks for your time. I have a few questions I would like to ask regarding the injuries you sustained recently that required you to be admitted to the Gold Coast Hospital.

#

**1.** To start off, can you please list the injuries you sustained that required you to be admitted to hospital on ………………… (Insert date here before interview).

____________________________________________________________________________________________________________________________________________________________________________________________________________________________________________________________________________________

**2.** Do you recall having a tertiary survey performed on you – that is a full head to toe review on the ward looking for all injuries you may have sustained?

❒ Yes

❒ No

❒ Can’t recall

**2a.** If answered ***YES*** to above, were any new injuries detected?

❒ Yes

❒ No

❒ Can’t recall

**2b.** If answered ***YES*** to above, what were these additional injuries?

_______________________________________________________________________________________________________________________________________________________________________________________________________________

**3.** After being discharged from hospital, have any other injuries been detected?

❒ Yes

❒ No

❒ Can’t recall

**3a.** If answered ***YES***, what were these?

| **Injury** | **Treatment?**  **(no Tx, splint, operation, other)** | **Referral**  **(Yes/No, if yes, to who?)** |
| --- | --- | --- |
| 1. |  |  |
| 2. |  |  |
| 3. |  |  |
| 4. |  |  |
| 5. |  |  |

**3b. After being discharged from hospital,** did you require further medical review?

❒ Yes

❒ No

If answered ***YES,*** did you… (can tick more than one)

❒ Yes – saw GP

❒ Yes – re-present to ED

❒ Yes – readmitted to hospital

❒ Yes – other _____________

**The next few questions are about how you were functioning PHYSICALLY before your recent injury that required hospital admission.**

**4.** Have you returned to the level of physical functioning for daily activities that you had before your injury?

❒ Yes, completely

❒ Yes, almost back to normal

❒ No, difficulty with some activities

❒ No, difficulty with most activities

❒ No, Unable to function at all

**4a.** If answered ***YES***… What do you put this down to? (prompt via open question)

❒ Injuries were minor

❒ Good Physiotherapy/Rehabilitation

❒ Good pain relief

❒ Other and detail below

__________________________________________________________________________________________________________________________________________

**4b.** If answered ***NO***… What do you put this down to (prompt via open question)?

❒ Injuries are severe/likely permanent

❒ Ongoing physio/rehabilitation need

❒ Nerve/muscle damage

❒ In plaster cast

❒ Other and detail below

_____________________________________________________________________

**5.** Before the injury, did you go to:

❒ School

❒ University

❒ Work

❒ Other ______

**5a.** If working, approximately how many hours each week did you work?

______ hours/week

**5b.** After your injury are you now back at:

School University

❒ YES ❒ YES

❒ NO ❒ NO

❒ N/A ❒ N/A

Work Other ______

❒ YES ❒ YES

❒ NO ❒ NO

❒ N/A ❒ N/A

**5c.** If working, Now, approximately how many hours each week do you work?

______ hours/week

**6.** Since your discharge from the hospital, please list whether you required any follow up and please specify who referred you for this follow up.

**- Physiotherapy** ❒ YES ❒ No ❒ Not required

Referred by:

❒ Hospital staff

❒ Self-referral

- Regular GP
- After-hours service

**- GP follow up** ❒ YES ❒ No ❒ Not required

Referred by:

❒ Hospital staff

❒ Self-referral

- Regular GP
- After-hours service

**- Occupational therapy** ❒ YES ❒ No ❒ Not required

Referred by:

❒ Hospital staff

❒ Self-referral

- Regular GP
- After-hours service

**- Nurse visit (eg Home team)**  ❒ YES ❒ No ❒ Not required

Referred by:

❒ Hospital staff

❒ Self-referral

- Regular GP
- After-hours service

**- Dietician** ❒ YES ❒ No ❒ Not required

Referred By:

❒ Hospital staff

❒ Self-referral

- Regular GP
- After-hours service

**- Psychologist** ❒ YES ❒ No ❒ Not required

Referred by:

❒ Hospital staff

❒ Self-referral

- Regular GP
- After-hours service

**- Other** _________________ ❒ YES ❒ No ❒ Not required

Referred By:

❒ Hospital staff

❒ Self-referral

- Regular GP
- After-hours service

**The next statements are about managing at home since your discharge and they ask you to consider which option best describes your experience. I will read each statement and the options out to you so that you can tell me which option is most appropriate.**

**7.** The hospital staff took my preferences and those of my family or caregiver into account in deciding ***what*** my health care needs would be when I left the hospital.

❒ Strongly Disagree

❒ Disagree

❒ Agree

❒ Strongly Agree

❒ Don’t know/ Don’t remember

**8.** When I left the hospital, I had a good understanding of the things I was responsible for in managing my health.

❒ Strongly Disagree

❒ Disagree

❒ Agree

❒ Strongly Agree

❒ Don’t know/ Don’t remember

**9.** When I left the hospital, I clearly understood the purpose for taking each of my

medications.

❒ Strongly Disagree

❒ Disagree

❒ Agree

❒ Strongly Agree

❒ Don’t know/ Don’t remember

❒ N/A Wasn’t on any medications when discharged

**10.** When I left hospital, I was able to manage my ***pain*** adequately?

❒ YES, all of the time

❒ YES, most of the time

❒ SOME of the time

❒ NO, mostly unable to manage pain

❒ NO, completely unable to manage pain

❒ I had no pain

**11.** When I left hospital, I was able to manage any equipment/dressings/splints/crutches without any difficulty?

❒ YES, all of the time

❒ YES, most of the time

❒ SOME of the time

❒ NO, mostly unable to manage equipment

❒ NO, completely unable to manage equipment

❒ Not applicable - didn’t have any equipment/dressings/splints when left hospital

**12.** When you left hospital, did you get your ***prescription*** for medications filled out at a chemist?

❒ Yes

❒ No

❒ Not applicable

**12a.** If ***NO***, what was the main reason not to fill prescription (open Question)?

❒ No time

❒ No money

❒ I forgot

❒ I did not think I needed the medication

❒ Other…..

**The last couple of questions are about our staff and your overall experience at the Hospital.**

**13.** How did you find the attitude of the staff in hospital overall?

❒ Very helpful

❒ Helpful

❒ Neutral

❒ Unhelpful

❒ Very unhelpful

**14.** Would you recommend Gold Coast Hospital to another person sustaining an injury the same as yours?

❒ Yes

❒ No

**This is the end of the interview. Thank you very much for your time and input.**

# **INTERVIEW – 6 months**

Thanks for your time again. Since our last interview, we would like to ask a few questions regarding your recovery after the injuries you sustained that required you to be admitted to the Gold Coast Hospital 6 months ago.

#

**1.** To start off, can you please list if any new injuries were detected after our first interview 1 month after discharged:

____________________________________________________________________________________________________________________________________________________________________________________________________________________________________________________________________________________

_____________________________________________________________________

**2. Since our last interview 1 month after discharge,** have you required further medical review for the injuries you sustained?

❒ Yes

❒ No

If answered ***YES,*** did you… (can tick more than one)

❒ Yes – saw GP

❒ Yes – re-present to ED

❒ Yes – readmitted to hospital

❒ Yes – other _____________

**The next few questions are about how you were functioning PHYSICALLY before your recent injury that required hospital admission.**

**3.** Have you returned to the level of physical functioning for daily activities that you had before your injury?

❒ Yes, completely

❒ Yes, almost back to normal

❒ No, difficulty with some activities

❒ No, difficulty with most activities

❒ No, Unable to function at all

**4a.** If answered ***YES***… What do you put this down to? (prompt via open question)

❒ Injuries were minor

❒ Good Physiotherapy/Rehabilitation

❒ Good pain relief

❒ Other and detail below

__________________________________________________________________________________________________________________________________________

**4b.** If answered ***NO***… What do you put this down to (prompt via open question)?

❒ Injuries are severe/likely permanent

❒ Ongoing physio/rehabilitation need

❒ Nerve/muscle damage

❒ In plaster cast

❒ Other and detail below

_____________________________________________________________________

**5.** Before the injury, did you go to:

❒ School

❒ University

❒ Work

❒ Other ______

**5a.** If working, approximately how many hours each week did you work?

______ hours/week

**5b.** After your injury are you now back at:

School University

❒ YES ❒ YES

❒ NO ❒ NO

❒ N/A ❒ N/A

Work Other ______

❒ YES ❒ YES

❒ NO ❒ NO

❒ N/A ❒ N/A

**5c.** If working, Now, approximately how many hours each week do you work?

______ hours/week

**6.** Since our last interview at 1 month after discharge from the hospital, please list whether you required any follow up and please specify who referred you for this follow up.

**- Physiotherapy** ❒ YES ❒ No ❒ Not required

Referred by:

❒ Hospital staff

❒ Self-referral

- Regular GP
- After-hours service

**- GP follow up** ❒ YES ❒ No ❒ Not required

Referred by:

❒ Hospital staff

❒ Self-referral

- Regular GP
- After-hours service

**- Occupational therapy** ❒ YES ❒ No ❒ Not required

Referred by:

❒ Hospital staff

❒ Self-referral

- Regular GP
- After-hours service

**- Nurse visit (eg Home team)**  ❒ YES ❒ No ❒ Not required

Referred by:

❒ Hospital staff

❒ Self-referral

- Regular GP
- After-hours service

**- Dietician** ❒ YES ❒ No ❒ Not required

Referred By:

❒ Hospital staff

❒ Self-referral

- Regular GP
- After-hours service

**- Psychologist** ❒ YES ❒ No ❒ Not required

Referred by:

❒ Hospital staff

❒ Self-referral

- Regular GP
- After-hours service

**- Other** _________________ ❒ YES ❒ No ❒ Not required

Referred By:

❒ Hospital staff

❒ Self-referral

- Regular GP
- After-hours service

**This is the end of the interview. Thank you very much for your time and input.**
